# Giant Panda Genomic Data Provide Insight into the Birth-and-Death Process of Mammalian Major Histocompatibility Complex Class II Genes

**DOI:** 10.1371/journal.pone.0004147

**Published:** 2009-01-07

**Authors:** Qiu-Hong Wan, Chang-Jun Zeng, Xiao-Wei Ni, Hui-Juan Pan, Sheng-Guo Fang

**Affiliations:** 1 College of Life Sciences, Zhejiang University, Hangzhou, People's Republic of China; 2 State Conservation Center for Gene Resources of Endangered Wildlife and the Key Laboratory of Conservation Genetics and Reproductive Biology for Endangered Wild Animals of the Ministry of Education, Hangzhou, People's Republic of China; Université de Toulouse, France

## Abstract

To gain an understanding of the genomic structure and evolutionary history of the giant panda major histocompatibility complex (MHC) genes, we determined a 636,503-bp nucleotide sequence spanning the MHC class II region. Analysis revealed that the MHC class II region from this rare species contained 26 loci (17 predicted to be expressed), of which 10 are classical class II genes (1 DRA, 2 DRB, 2 DQA, 3 DQB, 1 DYB, 1 DPA, and 2 DPB) and 4 are non-classical class II genes (1 DOA, 1 DOB, 1 DMA, and 1 DMB). The presence of DYB, a gene specific to ruminants, prompted a comparison of the giant panda class II sequence with those of humans, cats, dogs, cattle, pigs, and mice. The results indicated that birth and death events within the DQ and DRB-DY regions led to major lineage differences, with absence of these regions in the cat and in humans and mice respectively. The phylogenetic trees constructed using all expressed alpha and beta genes from marsupials and placental mammals showed that: (1) because marsupials carry loci corresponding to DR, DP, DO and DM genes, those subregions most likely developed before the divergence of marsupials and placental mammals, approximately 150 million years ago (MYA); (2) conversely, the DQ and DY regions must have evolved later, but before the radiation of placental mammals (100 MYA). As a result, the typical genomic structure of MHC class II genes for the giant panda is similar to that of the other placental mammals and corresponds to BTNL2∼DR1∼DQ∼DR2∼DY∼DO_box∼DP∼COL11A2. Over the past 100 million years, there has been birth and death of mammalian DR, DQ, DY, and DP genes, an evolutionary process that has brought about the current species-specific genomic structure of the MHC class II region. Furthermore, facing certain similar pathogens, mammals have adopted intra-subregion (DR and DQ) and inter-subregion (between DQ and DP) convergent evolutionary strategies for their alpha and beta genes, respectively.

## Introduction

The major histocompatibility complex (MHC) of vertebrates is a highly polymorphic region that controls immune responses through presentation of pathogen-derived peptides to T cells [1]. The MHC superfamily includes two major families, class I and class II. Class I molecules primarily present cytosol-derived peptides to cytotoxic CD8+ T cells, whereas class II molecules mostly present peptides derived from exogenous molecules to helper CD4+ T cells. These two classes of MHC molecules also differ in the structure of their antigen-binding (AB) domains. The AB domain of class I molecules is composed of two subdomains encoded by exons 2 and 3. The functional class II molecule is a heterodimer consisting of an alpha gene-encoded α chain and a beta gene-encoded β chain. Thus, the AB domain of the class II molecules is also a paired heteromeric structure, half of which is encoded by exon 2 of the alpha gene and half by exon 2 of the beta gene.

As the perceived primary role of the MHC genes has evolved from its original transplantation histocompatibility function [2] to “the center of the immune universe” [3], the MHC locus has become one of the most intensely studied genomic regions in vertebrates. The first of these regions to be completely sequenced, mouse MHC (H2) and human MHC (HLA) [4]–[5], revealed that the class I and II families are linked to a third region (class III) and contain unrelated genes, together constituting a single gene complex. The MHC class II region was further subdivided into DR, DQ, DP, DO, and DM subregions. The DR, DQ, and DP genes encode classical class II molecules responsible for presenting foreign peptides and triggering immune responses, whereas the DO and DM genes encode non-classical class II molecules that serve as molecular helpers promoting peptide binding to classical class II molecules [6]. Each subregion contains different numbers of alpha and beta genes. Furthermore, the number of expressed genes and pseudogenes also varies greatly among subregions.

The difference in the number of alpha and beta genes has been attributed to an evolutionary birth-and-death process in which new MHC genes have been created by repeated gene duplication, with some duplicate genes subsequently being deleted or becoming nonfunctional through the accumulation of deleterious mutations [7]. This evolutionary process has also contributed to the large difference in the number of MHC genes between species within a given mammalian order [8]. A comparison of class I and II sequences from major vertebrate groups demonstrated that the class I loci underwent a faster birth-and-death process than did class II loci, making it difficult to establish orthologous relationships of class I-a genes among different mammalian orders [9]–[10]. In contrast, the greater longevity of class II genes has made it relatively easy to identify orthologous class II loci in different orders of mammals [10]–[11]. As a result, and in view of the pivotal role of MHC genes in the immune system and the birth-and-death characteristics of class II genes, the MHC class II genomic regions for several mammals, including cat [12], dog [8], cattle [13], and pig [14], were sequenced to identify a complete and ordered set of MHC class II genes and resolve MHC evolutionary history from a comparative genomics perspective.

The giant panda is one of the world's most endangered species, currently consisting of a population of approximately 1600 individuals [15]. Historically, the range of giant pandas has included southern and eastern China and extended to northern Burma and northern Vietnam; but habitat loss has restricted the giant pandas to six isolated mountain ranges of China [16]. Considerable effort, involving both *in situ* and *ex situ* conservation strategies, has gone into protecting this rare species. At present, the captive giant panda population is approximately 240 individuals [17]. However, studies on artificially bred giant pandas have demonstrated reduced infectious disease resistance and reproductive success, problems that have severely hampered the development of the captive giant panda population [18] and created an incentive to explore more effective conservation strategies.

In addition to the crucial role of MHC in the immune response, increasing evidence suggests that MHC genes strongly influence the rate of reproductive success by controlling mate selection [19]–[20] and mother-fetus bio-compatibility [21]–[23]. Consequently, understanding genetic aspects of MHC genes should greatly aid in efforts to protect the giant panda. In this pioneering effort, we present the results of genomic sequencing of the giant panda. To resolve the number and order of the MHC class II genes, we have determined a 636,503-bp nucleotide sequence spanning the entire MHC class II region. We have then compared this sequence to that of MHC class II loci from other placental mammals to explore the evolutionary history of this important gene cluster.

## Results and Discussion

### Genomic structure and content

**Figure 1:**
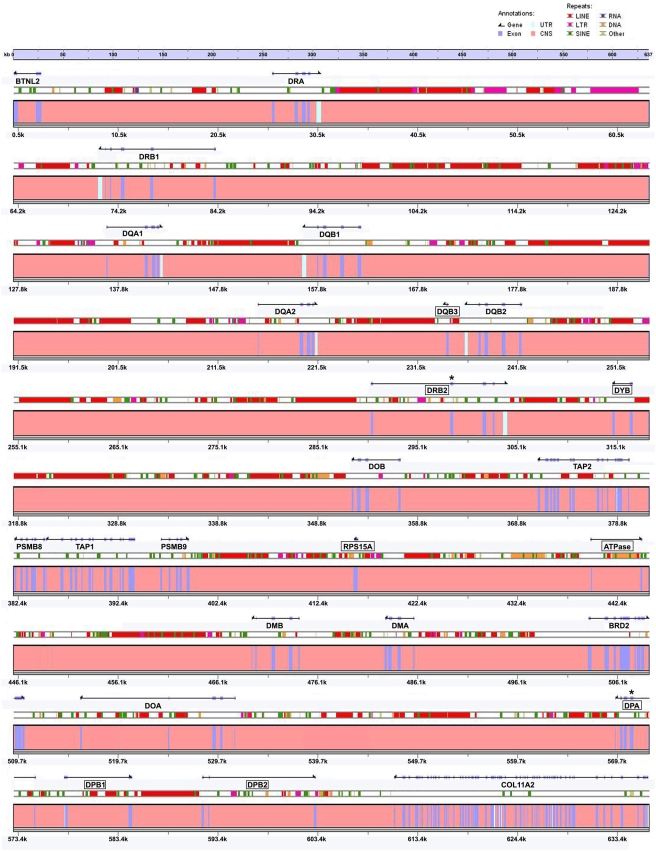
Genomic structure and content of giant panda MHC class II region. Boxes represent pseudogenes and the symbol “*” denotes pseudogenes that resulted from relatively recent mutation of functional genes.

A 636,503-bp nucleotide sequence spanning the giant panda MHC class II region was obtained by assembling contigs of 10 sequenced BAC clones. The giant panda MHC was designated *Aime-MHC* (*Aime* representing *Ailuropoda melanoleuca*), following the nomenclature system for the MHC of vertebrates [24]. A total of 26 genes were identified in *Aime-MHC* after repeat masking, GENESCAN prediction, and BLAST alignment. These were BTNL2, DRA, DRB1, DQA1, DQB1, DQA2, DQB3 ψ, DQB2, MAPRE1ψ, DRB2 ψ, DYB ψ, DOB, TAP2, PSMB8, TAP1, PSMB9, RPS15A ψ, ATPase ψ, DMB, DMA, BRD2, DOA, DPA ψ, DPB1 ψ, DPB2 ψ, and COL11A2 (Figure 1). Without repeat masking, GENESCAN actually predicted 52 loci based on the original 636,503-bp sequence, but the predicted results indicated that 23 of these were derived from repeats, and 5 non-repeat genes had incorrect exon boundaries that were mixed with intron or repeat sequences. Thus, the final gene prediction was achieved based on repeat-masked sequences. The original gene prediction suggested four novel transcripts, but after alignment with the NCBI nucleotide collection (nr/nt) database, these sequences were all found to correspond to homologous fragments in the MHC regions of other mammals, confirming that these were local sequences specific to the MHC region. None of these homologous loci was predicted to be expressed.

Of these 26 genes, those in the DR, DQ, and DP loci are common to placental mammals and belong to classical MHC class II gene families responsible for antigen presentation. The DO and DM genes encode non-classical MHC class II antigens related to “molecular helpers” that promote peptide loading onto classical class II molecules. The pseudogenes DRB2 and DPA were probably induced by relatively recent inactivating mutational events because both retained complete MHC genes that were normal except for a mutation resulting in a stop codon in exon 2 (DRB2) and the deletion of a codon in exon 2 (DPA). This showed that dysfunction of an MHC gene could arise as a result of those being a high spot for mutations and deletions. Other pseudogenes were severely reduced in size, usually comprising 1–2 exon fragments, probably resulting from large-scale deletions and gene recombination. The giant panda MHC class II region presented a linked gene cluster containing DOB, TAP2, PSMB8, TAP1, PSMB9, DMB, DMA, BRD2, and DOA. This gene group is present in this identical order in other sequenced mammals, including humans [5], cats [12], dogs [8], cattle [13], pigs [14], and mice [4]. We designated this DOB∼TAP2∼PSMB8∼TAP1∼PSMB9∼DMB∼DMA∼BRD2∼DOA gene cluster as the DO_box. Of these genes, PSMB encodes a proteasome subunit involved in cleaving peptides for presentation by class I antigen, the TAP gene product is a transporter for antigen processing, and BRD2 codes for a mitogen-activated nuclear kinase. Also in this cluster was BTNL2, which is a butyrophilin II-like gene of the immunoglobulin superfamily, and RPS15A ψ and ATPase ψ, which are pseudogenes that once encoded ribosomal protein S15a and vacuolar ATPase.

**Figure 2:**
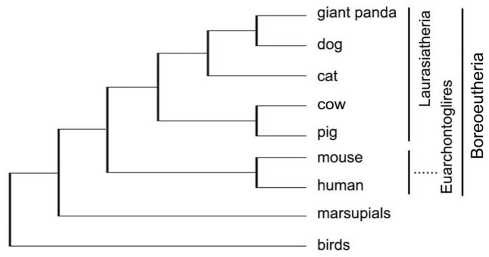
Phylogenetic relationships among the seven species sampled (modified from the phylogenetic tree of Springer et al. [33]).

There were two genes in this region of the giant panda genome, MAPRE1ψ and DYB ψ, that we would not have expected to find. Despite being a pseudogene, MAPRE1 ψ was detected in the Aime-MHC, but was absent in the MHC region of all the other mammals sequenced so far. The MAPRE1 (Microtubule-associated protein, RP/EB family, member 1) molecule is responsible for regulation of microtubule dynamic instability. In other mammals, the MAPRE1 gene is located in a different chromosome from that which harbors the MHC genes. For example, the MHC regions of human, dog, cow, pig, and mouse are located in chromosomes 6, 12, 23, 7, and 17, respectively, but their corresponding MAPRE1 genes are found on chromosomes 20, 1, 13, 17, and 2. This suggests that the giant panda's MAPRE1 ψ pseudogene has been transposed from another chromosome to the chromosome 9q region where the MHC is located [25]. The functional DY subregion normally contains a pair of DYA and DYB genes, which were thought to be nonclassical MHC class II loci [26] specific to ruminants [27]. The appearance of giant panda DYB ψ, albeit a pseudogene, suggests that the DY subregion is not strictly ruminant-specific, but was once shared by members of the Laurasiatheria superorder, including Ruminantia and Carnivora (Figure 2).

### Genomic comparisons

**Figure 3:**
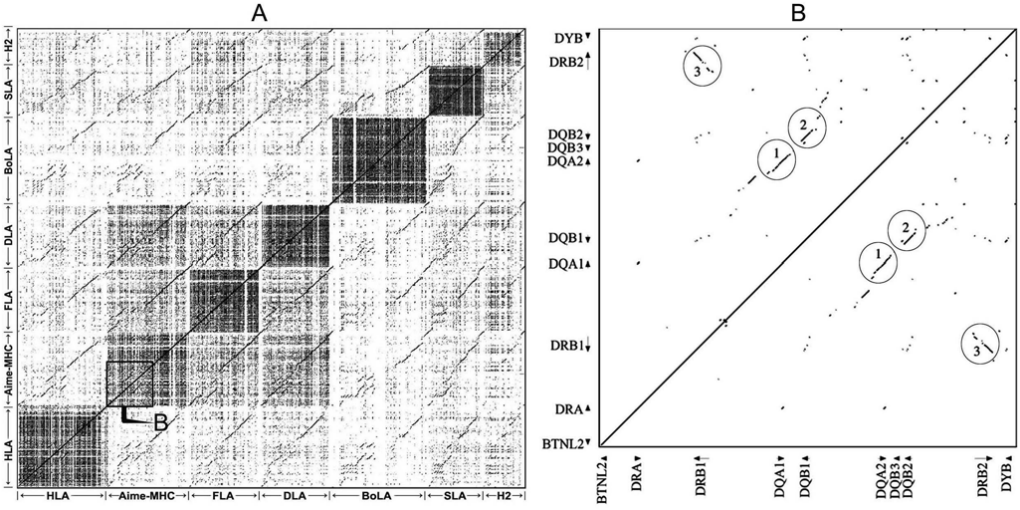
Dot plots of genomic MHC class II regions from seven species (A) and from the giant panda (B). The plots in A and B were produced by LBDot and Pipmaker, respectively.

Dot-plot analyses of the class II regions of *Aime-MHC*, HLA, FLA, DLA, BoLA, SLA, and H2 showed that the mammalian class II MHC retained multiple syntenic conserved segments, with the Carnivora MHC fragments of *Aime-MHC*, FLA, and DLA presenting the highest genomic similarities (Figure 3A). The self-sequence dot plot of the giant panda MHC class II indicated three large intra-MHC repeat segments corresponding to DQA1–DQA2, DQB1–DQB2, and DRB1–DRB2 pairs (Figure 3B). Additionally, the intraspecific dot plot of the giant panda yielded some small homologous plots (Figure 3B), which were probably giant panda- or MHC-region-specific repeats because repeat masking based on Carnivora common repetitive elements failed to reveal them before dot plotting.

**Figure 4:**
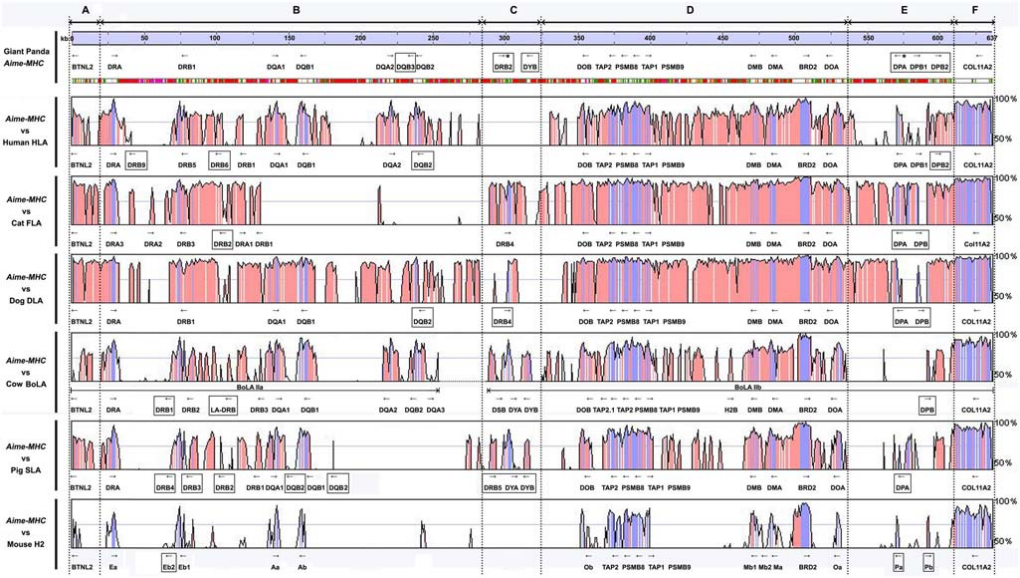
A VISTA plot of genomic comparisons for the seven species sampled. Boxes represent pseudogenes and the symbol “*”denotes pseudogenes that resulted from relatively recent mutation of functional genes. The color codes are as described in Figure 1.

The genomic organizations of the class II genes of *Aime-MHC*, HLA, FLA, DLA, BoLA, SLA, and H2 were compared using Multi-PipMaker and VISTA, both of which produced similar global genomic alignments. The VISTA plot (Figure 4) revealed that, despite a deficiency in the DQ subregion of FLA the Carnivora's FLA and DLA class II MHC had large segments that were homologous to those of the *Aime-MHC*, as shown in pair-wise dot plots (Figure 3). It also showed that the mammalian MHC class II region could be divided into six parts, including three conserved A, D, and F segments, and three highly variable B, C, and E segments. A, D, and F comprise BTNL2, the DO_box, and COL11A2, respectively, while B, C, and E correspond to DR1-DQ, DR2-DY, and DP subregions, respectively (Figure 4). The major differences among *Aime-MHC*, HLA, FLA, DLA, BoLA, SLA, and H2 were in the genomic length and content of the highly variable regions B, C, and E (Table 1 and Figure 4), a variability that was attributed to differences in alpha and beta gene birth-and-death process in DR, DQ, DY, and DP subregions (Figure 4). The cat MHC region lost the entire DQ subregion but expanded the DR subregion, resulting in the longest Carnivora BTNL2-DR1 region (225,849 bp; Table 1). The human HLA has developed into a MHC complex (∼702 kb long; Table 1) containing multiple suites of DR, DQ, and DP genes. It is also the only complex so far identified with two functional DPA and DPB genes (Figure 4). Similarly, the cow BoLA region has evolved into several sets of DR, DQ, and DY genes and possesses a pair of functional DYA and DYB genes (Figure 4). Both the expansion of DR and DQ subregions and the complement of DY genes in cow BoLA caused BoLA to become the longest mammalian MHC class II region of approximately 753,296 bp, after excluding the 17 cM gap (Table 1 and Figure 4).

**Table 1:** Comparisons among different subregions of the MHC class II regions from the seven species sampled. The numbers mean the length of genomic DNA.

		BTNL2-DR1	DQ	DR2-DY	DO-Box	DP-COL11A2	All
Giant panda		136,593 bp	153,776 bp	61,765 bp	179,235 bp	105,134 bp	636,503 bp
Cat		225,849 bp	/	55,602 bp	187,502 bp	92,002 bp	560,955 bp
Dog		121,917 bp	138,899 bp	17,685 bp	190,074 bp	84,688 bp	553,263 bp
Cow	II a	198,183 bp	130,646 bp				328,829 bp
	II b			167,554 bp	202,581 bp	54,332 bp	424,467 bp
Pig		146,035 bp	41,065 bp	45,546 bp	144,890 bp	49,680 bp	427,216 bp
Human		232,383 bp	175,758 bp	/	196,319 bp	97,641 bp	702,101 bp
Mouse		70,287 bp	48,759 bp	/	146,534 bp	50,399 bp	315,979 bp

A comparison across HLA, FLA, DLA, BoLA, SLA, and H2 showed that, although the *Aime-MHC* did not extend its DR subregion and retained only relic DP pseudogenes, its DQ subregion has expanded and four functional DQ loci survived the MHC gene death process (Figure 4), resulting in a total length of 636,503 bp (Table 1). In contrast, the cat FLA completely lacks the DQ subregion and the dog DLA retains expression of only a pair of DQA and DQB loci (Figure 4). The cow BoLA region was shown to possess the maximum number of expressed DQ members (five loci). The ongoing horse genome-sequencing project indicates that this herbivore might also have retained two or three pairs of functional DQ genes (NCBI *Equus caballus* Build 1.1). Collectively, we speculate that a high number of DQ loci could bear a relationship to the herbivorous habit. The identification of four functional DQ genes in the giant panda, which has evolved from a carnivorous habit to a diet of bamboo, is consistent with this interpretation. Thus, these genes were probably beneficial in the dietary adaptation of giant pandas.. The most striking characteristic in the VISTA-based genomic comparisons is the absence of the entire DR2-DY region in human HLA and mouse H2 (Figure 4). This contrasts sharply with the MHC regions of the other six mammals studied, all of which contained the DR2-DY region; however, only in ruminant cattle were all of these genes functional (Figure 4). In a previous study, Zeng et al. [28] concluded that the mammalian MHC class II region had the genomic structure: BTNL2∼DR∼DQ∼DOB∼DM∼DOA∼DP∼COL11A2. However, our comparative genomic study revealed that the DY subregion was not specific to ruminants, suggesting instead that the DY subregion might have been present in the genomic structure of ancestral mammalian MHC class II genes. The resolution of the ancestral MHC class II structure requires an estimate of the time at which DY genes arose in order to judge whether Euarchontoglires, such as humans and mice, lost the DY subregion, or Laurasiatheria, such as carnivores and herbivores, gained the DY genes after the separation of Euarchontoglires and Laurasiatheria.

### Phylogenetic tree and divergence time estimation

The above results highlight possible traditional misunderstandings of the mammalian genomic structure of the MHC class II region. Moreover, a recent study reported a completed opossum MHC genome sequence [29], providing a suite of MHC genes from marsupials. As a result, there is a need to reconstruct phylogenetic trees to distinguish the relationships among alpha and beta genes in different taxa. In our phylogenetic analysis, we excluded all pseudogenes because of their distorted exon content and evolutionary rates, and collected as many expressed alpha and beta genes as possible, including all those that were locus-defined and identified by genomic sequencing. The Bayesian-based and MP-based methods yielded phylogenetic trees with similar topology, so only the Bayesian phylogenetic trees are depicted.

**Figure 5:**
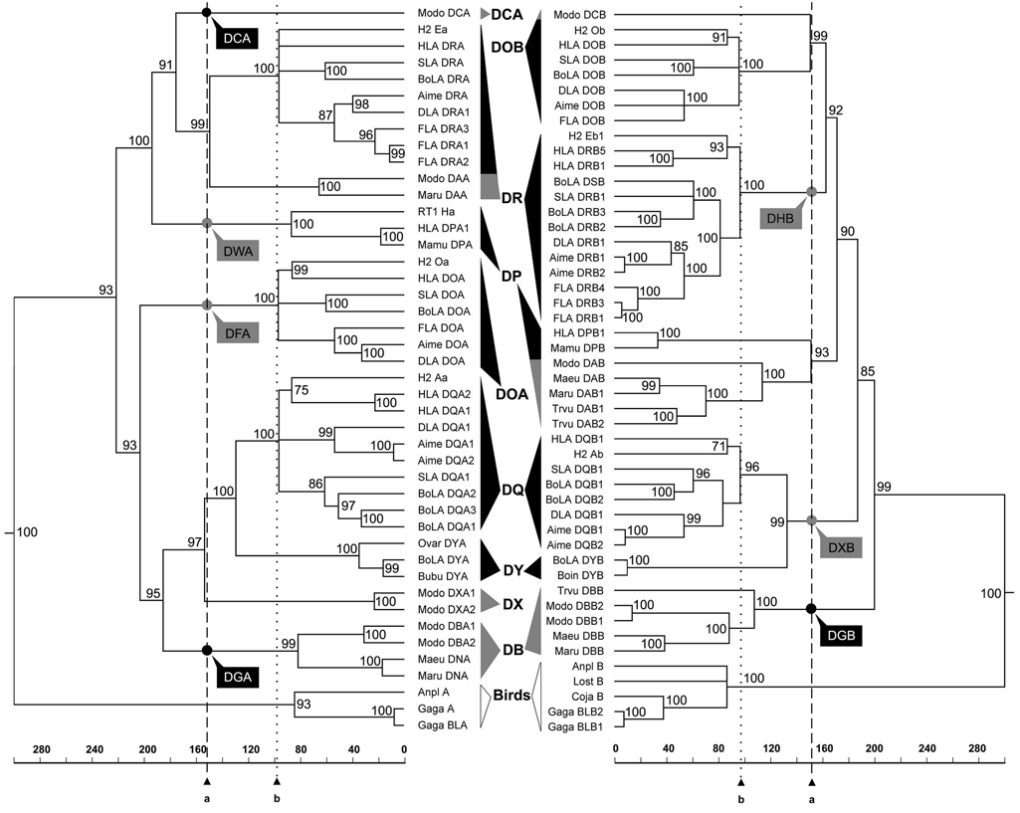
Molecular timescale (in millions of years) for the phylogenetic tree based on exons 3–4 of alpha and beta genes from marsupials (in gray) and placental mammals (in black). The *Aime*-DRB2 was included in the tree because of its critical position in the VISTA plot and the fact that its coding regions were relatively intact. The DMA and DMB genes were used as outgroups in constructing trees (data not shown). Triangles and rectangles represent gene clusters and lost genes, respectively; dashed and dotted lines represent the marsupial-placental and intra-Boreoeutheria divergences, respectively.

To exclude effects of the highly variable exon 2, whose variation was driven by pathogen variation, we first built a phylogenetic tree using the sequences of exons 3–4. The resulting tree showed that the DM genes clustered at the highest level, consistent with the fact that the DM genes developed before the split between birds and mammals [30]. Hence, the DMA and DMB genes were used as outgroups in constructing alpha and beta gene trees. The phylogenetic tree based on exons 3–4 had a very similar topology to that of the phylogenetic tree constructed using the different taxa sampled (Figure 5 and Figure 2). This made it possible to use the taxon-divergence time constraints for the splits between birds and mammals (>300 million MYA), marsupials and placental mammals (100–178 MYA), Laurasiatheria and Euarchontoglires (94–100 MYA), Feliform and Caniform (50–63 MYA), mouse and human (82–91 MYA), and cow and pig (58–65 MYA). The phylogenetic tree revealed four pairs of orthologous loci between marsupials and placental mammals with bootstrap values higher than 90 (Figure 5), namely, DXA and the ancestors of DQA-DYA, DAA and DRA, DCB and DOB, and DAB and DPB. Although we adopted a relaxed time range for the split between marsupials and placental mammals that incorporated different results from fossil and genetic data [31]–[34], both the alpha and beta trees supported the interpretation that marsupials diverged from placental mammals ∼150 MYA (Figure 5), in good agreement with the fossil record (143–178 MYA; [31]). The radiation time of placental mammals remains controversial, but a crude estimation of >100 MYA has been suggested [32]. In this study, the placental mammals sampled were all from Boreoeutheria fauna, whose members diverged at a relatively definite date, approximately 94–100 MYA [33]–[34]. As a result, multiple sets of orthologous loci from the giant panda, human, cat, dog, cow, pig, and mouse used the time constraint of 94–100 MYA for the DOA, DQA, DRA, DOB, DRB, and DQB gene clusters (Figure 5). MULTIDIVTIME dated the divergence of these mammals to 96–97 MYA according to estimates based on the results of different alpha and beta genes (DOA: 97.0 MYA; DQA: 96.5 MYA; DRA: 96.4 MYA; DOB: 96.0 MYA; DRB: 96.8 MYA; and DQB: 96.9 MYA). They suggest that the DR, DP, DO, and DM regions developed prior to the divergence of marsupials and placental mammals from a common ancestor (150 MYA), whereas the ancestral MHC alpha and beta genes evolved into the DQ and DY loci approximately 130 MYA (Figure 5); that is, before the intra-Boreoeutheria divergence (100 MYA), but after the marsupial-placental split (150 MYA). This indicates that the DY genes were lost in human and mouse rather than being gained in other mammals. Consequently, the *Aime-MHC* organization pattern, namely BTNL2∼DR1∼DQ∼DR2∼DY∼DO_box∼DP∼COL11A2, may typify mammalian MHC class II regions. In contrast to previous studies [10]–[11], which grouped the DQB and DPB regions together, our phylogenetic tree yielded separate branches comprising DPB of mammals and DAB of marsupials, and then clustered these with the major clade containing DOB and DRB genes (Figure 5).

**Figure 6:**
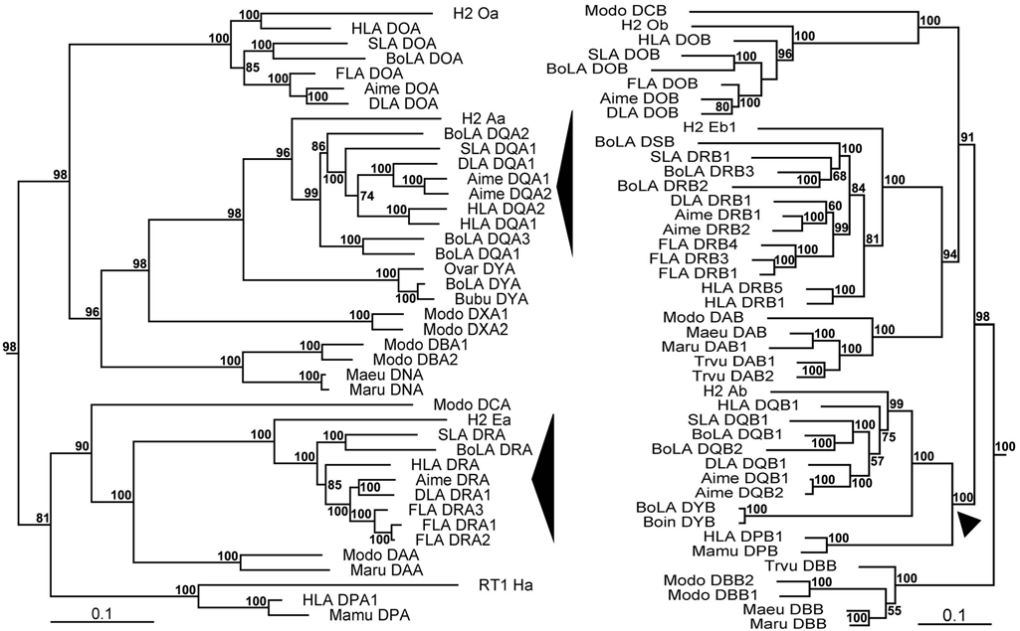
Phylogenetic trees based on all exons of alpha and beta genes from marsupials and placental mammals. The *Aime*-DRB2 was included in the tree because of its critical position in the VISTA plot and the fact that its coding regions were relatively intact. The DMA and DMB genes were used as outgroups in constructing trees (data not shown). Black triangles represent predicted evolutionary branches that are different from those in Figure 5.

To determine whether inclusion of the antigen presentation region (i.e., exon 2) would change the topology of the gene tree, we constructed another phylogenetic tree based on all exons. As expected, the new tree of beta genes grouped the DPB together with the DQB loci, but the topology of other genes was very similar to that of the exon 3–4–based tree (Figure 6). One difference, however, was that the new alpha-gene tree displayed intra-clade rearrangements relative to the former alpha tree, especially in the DQA and DRA clusters (Figure 6). In the former alpha tree, the intra-clade branches of DOA, DQA, and DRA clusters corresponded well to the taxonomic relationships of Euarchontoglires and Laurasiatheria (Figure 5 and Figure 2). However, with the exception of the DOA genes, human alpha genes were reassigned to the Laurasiatheria clade in the newly built tree (Figure 6). Exon 2 of MHC class II alpha and beta genes (except for nonclassical DO and DM loci) is a highly variable domain, and its variation is driven by continual competition among pathogen variants and host-pathogen co-evolution. Accordingly, the contrasts between the two trees (Figure 5 and Figure 6) indicate that mammals exposed to certain similar pathogens adopted intra-subregion (DR and DQ) and inter-subregion (between DQ and DP) convergent evolutionary strategies for their alpha and beta genes, respectively. Furthermore, the true phylogenetic relationships of MHC class II genes should depend on the sequences of exons 3–4, which are not involved in antigen presentation.

### Birth-and-death of MHC class II genes

The phylogenetic trees optimized by timescale provided a straightforward depiction of the MHC class II genes birth-and-death process (Figure 5). For example, the marsupials' DCA, DBA, and DBB genes and the placental mammals' DPA, DOA, DRB, and DQB-DYB genes diverged from a common marsupial-placental ancestor but orthologous DCA, DBA, and DBB placental and DPA, DOA, DRB, and DQB-DYB loci were not detected (Figure 5; for a convenient description, we assigned the descriptors DGA and DGB to the ancestral placental genes of DBA and DBB, and allocated the DWA, DFA, DHB and DXB to the ancestral opossum genes of DPA, DOA, DRB and DQB-DYB, respectively.). Thus, the placental mammals lost ancestral DCA, DGA (orthologous to DBA), and DGB (orthologous to DBB) genes whereas the opossum lost the DWA (orthologous to DPA), DFA (orthologous to DOA), DHB (orthologous to DRB), and ancestral DXB (orthologous to DQB-DYB) genes (Figure 5). The ancestral DX genes spawned two offspring families (DQ and DY subregions) in placental mammals approximately 130 MYA (Figure 5). In addition to birth and death of gene members, another process that molded MHC class II gene phylogeny is functional conversion. The DCA and DCB genes, which were paired in the opossum [29], went their separate ways in the mammals – DCA died off and DCB genes evolved into DOB genes (Figure 5). The DAA and DAB genes, also thought to be paired in marsupials, developed into DRA and DPB, respectively, in placental mammals (Figure 5). DB is the most active subregion in the opossum, but all DB genes ceased to exist in placental mammals (Figure 5).

**Figure 7:**
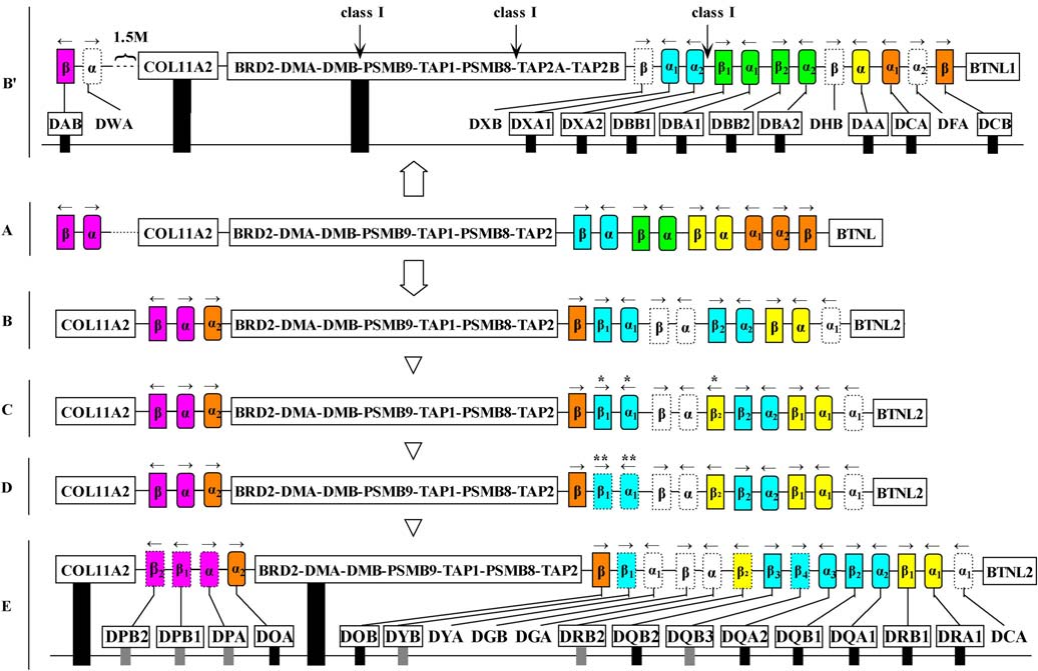
Hypothetical scenario for the birth and death of giant panda MHC class II genes. A, B', B, C, D, E depict the ancestral MHC structure of Theria, opossum, Boreoeutheria, Laurasiatheria, Carnivora and giant panda, respectively. The opossum MHC structure was modified from Belov et al. [29]. * denotes genes that are pseudo in Euarchontoglires and ** shows those genes that are functional in ruminants. The black and gray rectangles represent respectively functional and pseudogenes in the opossum and giant panda. The colour boxes and arrows indicate ancestral MHC genes and their orientation. The dotted colour and blank symbols reflect that the ancestral genes become pseudo genes and absent ones, respectively.

On the basis of the foregoing account, we created a schematic depiction of the mammalian MHC class II genes birth-and-death process (Figure 7). Incorporating the MHC genomic structure of the opossum and placental mammals, we propose a therian ancestral MHC organization with six alpha genes and five beta genes (Figure 7A). These subsequently developed into DO, DR, DQ-DY, and DP in placental mammals (Figure 7B–E) and DC, DA, DB, and DX in marsupials (Figure 7B'). Genes orthologous to DB loci were not detected in any of the Boreoeutherian animals sampled here, leading us to conclude that the ancestral DB alpha and beta genes had been lost in the Boreoeutherian ancestral MHC (Figure 7B). The phylogenetic tree also revealed that the evolution of ancestral MHC class II genes into DQ and DY subregions predated the intra-Boreoeutherian divergence, thus marking the presence of a gene duplication event in the Boreoeutherian ancestral MHC (Figure 7B). Because the giant panda, cat, dog, cow, and pig all possessed a DRB gene adjacent to the DY subregion (Figure 4), we included a duplicate DRB gene in the Laurasiatherian ancestral MHC (Figure 7C). Of the three Carnivora representatives – giant panda, dog, and cat – only the panda retained the relic DYB pseudogene (Figure 4). From this we infer that the DY loci had become pseudogenes in the Carnivora-ancestral MHC (Figure 7D). Considering that the giant panda possesses a relatively intact pseudo-DPA locus and exhibits a one-time duplication of the DPB gene, we conclude that the function of the panda's DP subregion was lost during the evolution of the panda instead of representing an event in the evolutionary history of the Carnivora-ancestral MHC. The MHC class II region of the giant panda ultimately evolved into its current species-specific genomic structure after losing the entire DYA locus, expanding the DQ subregion, and extinguishing the duplicate DRB gene (Figure 7E).

The birth of new MHC class II genes is rooted in genome fragment duplications, which are probably triggered by long interspersed nuclear elements (LINEs) [35]. A statistical analysis of repetitive elements in the MHC class II regions of giant panda, cat, dog, cow, pig, human, and mouse showed that each species had a characteristic distribution of repetitive elements (Table 2). The *Aime-MHC*, FLA and DLA of Carnivora contained the highest number of LINEs, whereas the HLA and H2 of Euarchontoglires had the most LTRs (Table 2). SINEs and LINEs were by far the most common repetitive elements of Cetartiodactyla BoLA and SLA, which had the highest content of SINEs among all MHC regions compared (Table 2). A comparison of repeat content between the entire MHC class II region and the DR1-DQ-DR2-DY region revealed a clearly evident increase in LINE and LTR contents in the gene-rich DR1-DQ-DR2-DY region (Table 2). In sharp contrast to LINEs and LTRs, SINEs and DNA transposon content were generally observed to decrease in the DR1-DQ-DR2-DY region, suggesting that both LINEs and LTRs are associated with the MHC class II gene fragment duplication process. In the HLA, the LINE content of various subregions was similar to the overall LINE content, but the frequency of LTRs was elevated in the gene-rich DR∼DQ region (Table 2), suggesting that, in certain species, LTRs may play a role in the duplication of MHC class II genes.

**Table 2:** Comparisons among different repetitive elements in MHC class II regions from the seven species sampled.

	SINEs		LINEs		LTRs		DNA transposon		All types	
	A∼F	B∼C	A∼F	B∼C	A∼F	B∼C	A∼F	B∼C	A∼F	B∼C
Giant panda	7.15	5.57	**30.74**	38.40	3.77	4.86	2.74	1.65	45.66	51.72
Cat	10.22	8.57	**26.94**	31.38	4.70	7.31	2.16	1.03	45.74	50.54
Dog	9.39	6.84	**31.33**	42.61	4.02	5.63	2.54	1.32	48.85	57.77
Cow (II a+II b)	**16.21**	16.32	19.43	21.52	2.57	2.17	1.87	1.68	41.47	42.68
Pig	**18.21**	15.84	12.44	15.34	1.65	2.14	2.19	1.26	36.24	36.22
Human	10.10	9.94	25.38	25.59	**12.40**	16.36	4.17	4.15	53.76	57.54
Mouse	11.61	8.03	4.48	8.42	**15.32**	17.74	0.50	0.70	34.59	37.75

A∼F corresponds to the subregions of MHC class II region in Figure 4. The maximum values are highlighted in bold.

## Materials and Methods

### BAC clones for sequencing

Construction of a sequence-ready BAC contig spanning the MHC class II region was previously described [28]. This contig is approximately 650-kb in length and includes the entire classical class II region and a part of the extended class II regions. In this study, only the classical class II region containing alpha and beta genes was sequenced. In total, ten BAC clones (504E11, 354C3, 826G2, 237E1, 1071G6, 206G5, 900H8, 692B2, 1561B8, and 1262B6) were sequenced.

### Preparation of BAC DNA and generation of shotgun libraries

A single BAC clone was used to inoculate 1 ml of LB containing 12.5 µg/mL chloramphenicol. After incubating at 37°C with shaking for 5–6 h, 1 µL of Copycontrol induction solution was added and the culture was incubated at 37°C with shaking for an additional 1–2 h. A 1-mL aliquot of the induced culture was transferred to 500 mL of LB containing 12.5 µg/mL chloramphenicol and then incubated overnight with shaking at 37°C. Ultrapure BAC DNA was isolated from the overnight, induced culture using the Qiagen Large-Construct Kit. High-molecular-weight BAC DNA was dissolved in 2 mL of TE buffer and stored at −20°C until use.

Shotgun libraries were constructed according to the protocol of Yuhki et al. [12], with modifications. Briefly, approximately 300 µL of ultrapure BAC DNA was sonicated to obtain sheared DNA fragments ranging from 1.5 to 3.5 kb in size. The randomly sheared DNA was end-repaired by incubating with 1.5 µL of Mung Bean Nuclease and then heated at 65°C for 5 min to inactivate the nuclease. The repaired DNA fragments were ligated into *Sma*I-digested pUC19 vector, and then used to transform competent DH5α cells. Plasmid DNA was extracted using the AxyPrep-96 Plasmid Purification Kit (Axygen) according to the manufacturer's protocol.

### Sequencing and assembly of shotgun clones

Plasmid DNA from shotgun clones was sequenced in both directions with M13 forward and reverse primers to achieve 7.2- to 8.8-fold coverage. The sequencing reactions were carried out using dye-terminator and dye-primer chemistry on an ABI Prism 3730x1 DNA analyzer (Applied Biosystems) by the sequencing group of Beijing Genomics Institute, and on a LI-COR Long Read IR^2^ 4200 DNA automatic sequencer (Li-Cor, NEN) by our research group.

For the sequences obtained from the ABI DNA analyzer, base calling and quality assessment were performed using PHRED [36]–[37]. The nucleotide sequences were first examined for contaminating vector and *E. coli* DNA with PHRAP (http://www.phrap.org) and the trimmed sequences were assembled by CONSED [38]–[39]. For the sequences derived from the LI-COR IR^2^ sequencer, base calling and quality control were carried out using E-seq (http://www.licor.com) and the sequence pre-processing and assembly were performed using the SeqMan module of the DNAStar software package. Sequence gaps were closed by primer walking or by sequencing PCR products that spanned small gaps [13]. The primers for closing gaps were designed from unique end-sequences from each assembly after first analyzing for Carnivora repetitive elements by RepeatMasker (http://www.repeatmasker.org).

### Sequence analysis and annotation

The finished genomic sequence of each BAC clone was analyzed by RepeatMasker (http://www.repeatmasker.org); GENESCAN was used to predict genes from the masked nucleotide sequence ([40]; http://genes.mit.edu). The predicted gene sequences were aligned against NCBI reference mRNA sequences using BLAST programs [41] to identify the boundaries of exons (i.e., to prevent contamination of coding regions by introns) and to distinguish expressed genes from pseudogenes. The GENESCAN-predicted peptide sequences were aligned against non-redundant protein sequences by BLASTP [42] to search for amino-acid homology. If a novel transcript was predicted but failed to yield homologous fragments in either BLAST search, it was realigned against the NCBI nucleotide collection (nr/nt) database to exclude an origin from local region-specific sequences. The 10 BAC-derived contigs were assembled into a complete nucleotide sequence for the giant panda MHC class II region and reanalyzed for predicted genes by GENESCAN to produce the final annotated genomic sequence.

### Genomic comparisons

For comparative analyses, we downloaded the complete MHC class II sequence from human (HLA: NT_007592), cat (FLA: AY152826, AY152828–31, AY152833, AY152834 and AY152836), dog (DLA: AJ63062–66), cow (BoLA), pig (SLA: BX323846, BX088590, BX323833, BX324144 and BX640585), and mouse (H2: AF050157, AF027865 and AF100956). With the exception of the bovine sequence, all sequences were derived from BAC clones. The MHC class II region of the cow was divided into two parts, BoLA-IIa and BoLA-IIb, the latter of which was inverted from the former to a position near the centromere [13] resulting in an intra-BoLA 17 cM gap [43]. The BoLA-IIa sequence was from the *Bos taurus* genome sequencing project (NCBI Btau 3.1; 21229140∼21568700), and the BoLA-IIb sequence was from AY957499. The MHC class II sequences from human, cat, dog, cow, pig, and mouse were assembled separately and trimmed to correspond to the beginning and end of the giant panda sequence. The annotated MHC class II region of the giant panda and the trimmed corresponding sequences from other mammals were aligned using LBDot [44], PipMaker ([45]; http://bio.cse.psu.edu/pipmaker), and the AVID algorithm [46], implemented in VISTA ([47]; http://genome.lbl.gov/vista).

### Construction of phylogenetic trees

A phylogenetic tree was constructed from predicted functional giant panda alpha and beta genes and the expressed loci identified from the completely sequenced MHC class II regions of the mammals under study. These loci included human HLA (NT_007592: DRA, DRB1, DRB5, DQA1, DQA2, DQB1, DPA1, DPB1, DOA, DOB, DMA, and DMB), cat FLA ([12]: DRA1, DRA2, DRA3, DRB1, DRB3, DRB4, DOA, DOB, DMA, and DMB), dog DLA (AJ63062–66: DRA, DRB1, DQA1, DQB1, DOA, DOB, DMA, and DMB), bovine BoLA (Btau 3.1 and AY957499: DRA, DRB, DRB3, DSB, DQA1, DQA2, DQA3, DYA, DYB, DOA, DOB, DMA, and DMB), pig SLA ([14]: DRA, DRB1, DQA1, DQB1, DOA, DOB, DMA, and DMB), and mouse H2 ([4]: Ea, Eb1, Aa, Ab, Oa, Ob, Ma, Mb1, and Mb2). All pseudogenes were excluded from the phylogenetic analysis because of the distorting effect of their atypical evolutionary rates and because many of their coding sequences were incomplete. To enhance the power of resolution of the phylogenetic trees, we supplemented several cDNA sequences from other mammals to compensate for deficiencies in the numbers of particular functional genes in our primary mammalian dataset. These added sequences included DPA from *Rattus norvegicus* (NM_001008848: RT1 Ha) and *Macaca mulatta* (AB250756: *Mamu*-DPA), DPB from *Macaca mulatta* (EF490966: *Mamu*-DPA), DYA from *Bubalus bubalis* (DQ188097: *Bubu*-DYA) and *Ovis aries* (NM_001123398: *Ovar*-DYA), and DYB from *Bos indicus* (AJ251358: *Boin*-DYB).

To completely resolve the evolutionary history of giant panda MHC class II genes, we also included some expressed alpha and beta genes from marsupials during phylogenetic tree construction. These included MHC class II genes from *Monodelphis domestica* (NCBI MonDom5 and opossum MHC browser http://bioinf.wehi.edu.au /opossum/seq/class_II.fa: *Modo*-DCA, *Modo*-DCB, *Modo*-DAA, *Modo*-DAB, *Modo*-DBA1, *Modo*-DBB1, *Modo*-DBA2, *Modo*-DBB2, *Modo*-DXA1, *Modo*-DXA2, *Modo*-DMA, and *Modo*-DMB); *Macropus rufogriseus* (U18109: *Maru*-DAA; U18110: *Maru*-DNA; M81624: *Maru*-DAB1; M81625: *Maru*-DBB); *Macropus eugenii* (L12123: *Maeu*-DNA); and *Trichosurus vulpecula* (AY271265: *Trvu*-DBB; AF312029: *Trvu*-DAB1; AF312030: *Trvu*-DAB2). Also included were MHC class II genes from birds, including *Gallus gallus* (AL023516: *Gaga*-DMA, *Gaga*-DMB1, *Gaga*-DMB2, *Gaga*-BLA, *Gaga*-BLB1, and *Gaga*-BLB2; DQ008588: *Gaga*-A), *Anas platyrhynchos* (AY905539: *Anpl*-A and DQ490139: *Anpl*-B), *Coturnix japonica* (AB110476: *Coja*-B), and *Lonchura striata* (L42334: *Lost*-B).

The analysis of alpha and beta genes from diverse mammals, marsupials, and birds was facilitated by first aligning sequences by translated amino acids to locate the positions of gaps in the corresponding codon positions, as implemented in Mega 4.0 [48]. The aligned sequences were subjected to Bayesian phylogenetic analyses with MRBAYES 3.1.2 [49]. Analyses were run with one cold chain and three heated chains for 500,000 generations. Amino-acid analysis was based on the POISSON model and nucleotide analysis adopted a codon-based GTR+G+I model of substitution. Maximum parsimony (MP) analysis was also performed by PAUP 4.0beta [50] for comparison of trees. The MP tree was produced using heuristic searches with tree-bisection-reconnection (TBR) branch swapping; node support was tested by bootstrapping 100,000 replicates.

### Estimation of divergence times

Divergence times were estimated using a multiple calibration Bayesian approach with the MULTIDISTRIBUTE program package version 09.25.03 [51]. This method applies a relaxed molecular clock that does not require the assumption of constant evolutionary rates among genes and lineages, and allows the use of prior constraints on divergence times. Molecular dating by MULTIDISTRIBUTE was achieved through three programs, PAML [52], ESTBRANCHES [53], and MULTIDIVTIME [51], [53]. Using the phylogenetic relationships among the major taxa depicted in Figure 2, we applied six age constraints to calibrate the molecular clock and estimate divergence times between clusters in subsequent gene trees. The time constraints included splits between (1) birds and mammals (>300 MYA; [54]–[55]), (2) marsupials and placental mammals (100–178 MYA; [31]–[34]), (3) two super clades (Laurasiatheria and Euarchontoglires) of placental mammals (94–100 MYA; [33]–[34]), (4) Feliform and Caniform (50–63 MYA; [33], [55]), (5) mouse and human (82–91 MYA; [33]–[34], [56], and (6) cow and pig (58–65 MYA; [32], [57]–[58]).
